# The experiences of people with liver disease of palliative and end‐of‐life care in the United Kingdom—A systematic literature review and metasynthesis

**DOI:** 10.1111/hex.13893

**Published:** 2023-10-19

**Authors:** Cathy J. Beresford, Leslie Gelling, Sue Baron, Linda Thompson

**Affiliations:** ^1^ Department of Nursing Science Faculty of Health and Social Sciences, Bournemouth University Bournemouth UK; ^2^ Lewis‐Manning Hospice Care Poole UK

**Keywords:** empowerment/disempowerment, end‐of‐life, experiences, liver disease, palliative care

## Abstract

**Background:**

Liver disease is a growing health concern and a major cause of death. It causes multiple symptoms, including financial, psychological and social issues. To address these challenges, palliative care can support people alongside active treatment, and towards the end of life, but little is known about the care experiences of individuals with liver disease in the United Kingdom. This review aimed to explore the palliative and end‐of‐life care experiences of people with liver disease in the United Kingdom.

**Method:**

A systematic review was conducted using a five‐stage process and following Preferred Reporting Items for Systematic Reviews and Meta Analyses guidelines. Searches were across Web of Science, Scopus, EBSCO and grey literature until 10 May 2023. The review was registered through International Prospective Register of Systematic Reviews (PROSPERO). NVivo 12.5 was used to facilitate data analysis (systematic review registration: PROSPERO CRD42022382649).

**Results:**

Of 6035 papers (excluding duplicates) found from searches, five met the inclusion criteria of primary research related to adults with liver disease receiving palliative and/or end‐of‐life care in the United Kingdom, published in English. Reflexive thematic analysis of the data was conducted. The themes identified were the experiences of people with liver disease of relating to healthcare professionals, using services, receiving support, and experiences of information and communication. These were connected by an overarching concept of disempowerment versus empowerment, with the notion of person‐centred care as an important feature.

**Conclusion:**

This review has found variations in the care experiences of people with advanced liver disease towards the end of life and an overall lack of access to specialist palliative care services. Where services are designed to be person‐centred, experiences are more empowering. Further research is needed but with recognition that it is often unclear when care for people with liver disease *is* palliative or end‐of‐life.

**Patient and Public Contribution:**

An online public involvement workshop was held on 18 April 2023 through Voice (2023). This included four people with liver disease and four carers to discuss the review findings and to design a qualitative research study to further explore the topic.

## 1 BACKGROUND

Liver disease is increasing in the United Kingdom, and it is a major cause of mortality worldwide, accounting for two million deaths annually.^1^ Data from the Office for National Statistics^2^ shows a 63.6% increase in the number of premature deaths in England from liver disease in the past 20 years, and it was the third leading cause of death in 2021 for those aged between 35 and 49 years.^3^ Around 60% of individuals affected are male and approximately 40% are female.^2^ The disease trajectory can be unpredictable,^4^ it is not always clear when someone is reaching the end of their life, and 20% of people on the liver transplant list die while they are waiting for transplantation.^5^ Active treatment may continue until shortly before a person dies because of uncertain prognosis, young age of individuals, and rapid decline in health.^6^ Advanced liver disease causes numerous symptoms and social and financial issues.^7^, ^8^

Palliative care is the supportive treatment that can be offered alongside active treatment to help with symptoms and the challenges of living with liver disease. The International Association for Hospice and Palliative Care^9^ defines palliative care as ‘the active holistic care of individuals across all ages with serious health‐related suffering due to severe illness, and especially of those near the end of life. It aims to improve the quality of life of patients, their families, and their caregivers’. It is commonly perceived that palliative care is synonymous with end‐of‐life care, but increasingly it is recognised that palliative care can be offered at any stage to ease symptoms and psychosocial issues for people with liver disease.^8^, ^10^ The concept of ‘parallel planning’ in liver care^8^ is an approach that acknowledges the need to actively manage complications, whilst also preparing people with liver disease, and their families, for potential deterioration in their health.

There are guidelines to support clinicians caring for people with end‐stage liver disease^11^, ^12^ and the American Association for the Study of Liver Diseases recently published a guidance document.^13^ However, healthcare professionals (HCPs) may not know when to refer people for palliative care or be confident discussing it with individuals.^14^ People with liver disease can be unclear about what palliative care is, tending to associate it with end‐of‐life and the loss of active therapy.^15^ The benefits of palliative care are well documented,^15^ but there is a lack of research focusing on the experiences of people with liver disease of palliative care. Similarly, a recent literature review found limited research about patient's perspectives on end‐of‐life care in liver disease and Das et al.^16^ recommended future studies focusing on their perspectives.

Socioeconomic factors significantly influence liver disease mortality and people living in the most deprived areas are more adversely affected, including higher rates of hospital admissions.^17^ National Health Service NHS Long Term Plan^18^ advocates stronger action for reducing health inequalities, including access to healthcare provision, and this review is being used to develop a research project that could help to improve the effective implementation of person‐centred care for people with advanced liver disease.

### 1.1 Aim

The aim of this systematic review was to understand more about the experiences of people with liver disease in palliative and/or end‐of‐life care.

## 2 METHODS

The Preferred Reporting Items for Systematic Reviews and Meta Analyses (PRISMA) checklist^19^ guided this systematic review report. This review explored the subjective experiences of people with liver disease; therefore, qualitative data was appropriate to answer the review question. The reviewers did not set out to exclude quantitative data, but it was apparent early on that qualitative evidence was required, which included mixed‐methods studies. A qualitative methodological approach was followed, including metasyntheses, which are systematic techniques to combine the in‐depth, rich qualitative research findings, to provide insights into the research question.^20^ A five‐stage method for rigorously reviewing the literature, as proposed by Wolfswinkel et al.,^21^ enabled a systematic process of theory development, providing insight and understanding into the experiences of people with liver disease of palliative and end‐of‐life care. This consisted of the following steps:

1. Define (the suitable data set)
2. Search (for the studies)
3. Select (refine the sample of studies to be reviewed)
4. Analyse (including open coding, axial coding, and selective coding)
5. Present (to show findings and insights, alongside key decisions made throughout the review process).

Public involvement (PI) was incorporated into this systematic review at Stage 5. The ACTIVE framework to describe stakeholder involvement in systematic reviews^22^ is Supporting Information: Appendix S1. The protocol for the systematic review was registered with the International Prospective Register of Systematic Reviews in accordance with PRISMA‐P guidelines (CRD42022382649).

### 2.1 Developing the research question

An initial check of the literature found that internationally, researchers have explored the experiences of people with advanced liver disease^22^ and transnational systematic reviews have revealed shortfalls in access to palliative care.^16^, ^24^, ^25^ However, health systems vary across the world and the experiences of people with liver disease may also differ because of this. For example, in the US individuals may have financial concerns around paying for their treatment, which are different to the United Kingdom. This systematic review forms part of a PhD project and is helping to shape a UK study with people who have advanced liver disease. Therefore, it was decided to focus on the United Kingdom. After scanning the literature and discussing with HCPs and people with liver disease, the review question was formed using the PEO format^26^: What are the experiences of adults with liver disease of palliative and end‐of‐life care in the United Kingdom?

Population—adults with liver disease.

Exposure—palliative and end‐of‐life care in the United Kingdom.

Outcome—experiences.

#### 2.1.1 Stage 1: Define

The initial selection criteria were decided before the searches were conducted, and a logbook was kept recording any changes to the criteria that were made throughout the review process.^21^ For example, when assessing papers against the selection criteria, it became apparent that to understand the experiences of people with liver disease, papers that did not include the perspectives and experiences of people with liver disease *from their point of view* should be excluded because such papers did not answer the research question. This meant that studies without some qualitative data were not included.

### 2.2 Inclusion criteria

Included papers were primary research related to adults with liver disease receiving palliative and/or end‐of‐life care in the United Kingdom, published in English.

### 2.3 Exclusion criteria

Publications about people without liver disease as their primary diagnosis were excluded, as were systematic and other types of reviews. Other exclusion reasons were as follows:

1. Participants <18 years
2. Not specific to end of life or palliative care, for people with liver disease.
3. No reference to the perspectives and/or experiences of people with liver disease *from their point of view*—the papers had to include the voices of people with liver disease. It became apparent early in the review that this excluded research that was purely quantitative.
4. Research outside of the United Kingdom.

#### 2.3.1 Stage 2: Search

Searches were conducted by the lead reviewer between January and February 2023 across Web of Science, Scopus and EBSCO platforms to search multiple databases simultaneously including CINHAL Complete and MEDLINE Complete. Cochrane and PROSPRO were also searched. Reference lists and citation searches of the included papers and relevant systematic reviews were conducted. Grey literature searching included Google Scholar, the British Liver Trust, and Electronic Theses Online Services (EThOS). The university librarian provided support to ensure a robust search with suitable key terms (Table 1).

**Table 1:** Search terms included.

Keyword	Liver N3 disease OR cirrho* OR Hepat* OR liver N3 cancer OR ascit*
And	“end of life” OR palliative OR dying OR die OR terminal* OR advanced OR “quality of life”
And	experience* OR perception* OR attitude* OR view* OR feeling* OR qualitative OR perspective

#### 2.3.2 Stage 3: Select

**Figure 1:**
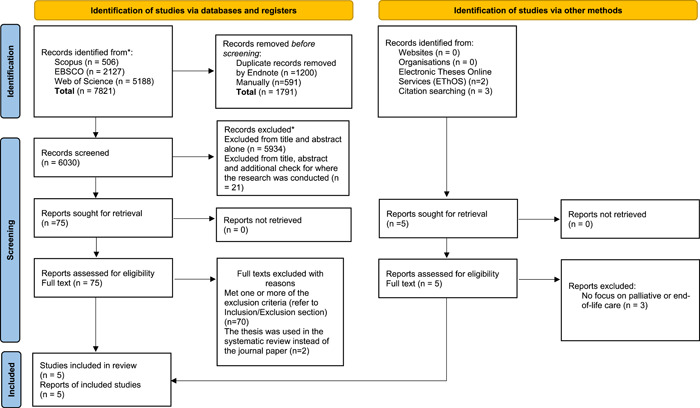
PRISMA 2020 flow diagram for new systematic reviews which included searches of databases, registers and other sources. *From*: Page MJ, McKenzie JE, Bossuyt PM, Boutron I, Hoffmann TC, Mulrow CD, et al. The PRISMA 2020 statement: an updated guideline for reporting systematic reviews. *BMJ*. 2021;372:n71. doi: 10.1136/bmj.n71. For more information visit: http://www.prisma-statement.org/. PRISMA, Preferred Reporting Items for Systematic reviews and Meta Analyses.

Figure 1 shows the Prisma 2020 diagram.^19^ A total of 7821 results were exported into Endnote. About 1791 duplicates were removed, and 6030 titles and abstracts were assessed by the lead reviewer against the selection criteria and in discussion with the rest of the review team at monthly meetings between January and March 2023. Total of 5934 were removed from the title and abstract alone, with a further 21 removed with additional checks as to where the research was conducted (to exclude non‐UK). Seventy‐five full texts were retrieved, of which initially 67 were excluded because they met one or more of the exclusion criteria. Three papers were found from citation searching and two theses from EThOS. Initially, the first reviewer included nine papers in the review based on the inclusion criteria. However, not all of these were clearly focusing on palliative or end‐of‐life care (although there were relevant issues). For example, Chivinge et al.^27^ was a service improvement project for individuals with ascites which is a poor prognostic indicator^28^ but not necessarily end‐of‐life. None of the individuals in Day et al.'s^29^ study (on experiences of nonmalignant ascites and its treatment) referred to their illness as palliative and care was not specifically end‐of‐life. Davis et al.^30^ provided a sociological critique of healthcare encounters of people with cirrhosis, but the focus was not end‐of‐life or palliative care. Both Kimbell and Hudson had journal papers published as part of their PhD research.^6^, ^31^ These were read by the review team, and it was agreed that for the systematic review, it would be more thorough to include the theses rather than the journal papers of these studies. Therefore, with careful consideration and discussion between the review team, the included papers were narrowed down to five.

### 2.4 Data extraction

Using Excel, a data summary chart (see Table 2) was created with an overview of the characteristics and findings of the included papers, including authors, date, title, the geographical location and setting of the research, the aim of the study, participants, methods, main findings, strengths/weaknesses, including ethical considerations, PI in the research, and the authors' recommendations. This was completed by the first reviewer (C. B.) in consultation with the other reviewers.

**Table 2:** Data summary chart.

Authors, date, title	Cooper M, Pollard A, Pandey A, et al. 2021. Palliative long‐term abdominal drains (LTADs) versus large volume paracentesis (LVP) in refractory ascites due to cirrhosis (REDUCe study): Qualitative outcomes.	Hudson, B., 2019. The Integration of Palliative Care into the Management of End Stage Liver Disease. Thesis (PhD).	Kimbell, B., 2014. Living, dying and caring in advanced liver disease: the challenge of uncertainty. Thesis (PhD).	Kimbell, B., Murray, S. A., Byrne, H., Baird, A., Hayes, P. C., MacGilchrist, A., Finucane, A., Brookes Young, P., O'Carroll, R. E., Weir, C. J., Kendall, M. and Boyd, K., 2018. Palliative care for people with advanced liver disease: A feasibility trial of a supportive care liver nurse specialist.	Quinn, S., Campbell, V. and Sikka, K., 2017. Sooner rather than later: early hospice intervention in advanced liver disease.
Country of study	England	England	Scotland	Scotland	England
Geographical area	Two sites: Brighton and Worthing	Bristol	South‐east Scotland	Edinburgh (from the authors' details)	Essex
Study setting	Hospital and community	University Hospitals Bristol day‐case unit	Inpatient liver unit	Tertiary gastroenterology/hepatology hospital ward	A hospice and the hepatology team based at a district general hospital.
Aims of the study	To explore and contrast experiences/perceptions/care pathways of people with refractory ascites due to end‐stage liver disease randomised to either palliative LTADs (allow home drainage) versus LVP (hospital drainage).	The thesis is made up of four component studies. *The component study with people with liver disease, and their carers, is relevant to answering this systematic review question*. The aim was to understand the palliative care needs of patients with end‐stage liver disease, and their carers, ascertain how existing services meet these needs, and explore the attitudes of patients and carers towards palliative care.	To broaden our understanding of the experience of living and dying with advanced liver disease. Specifically, it sought to explore the dynamic physical, psychosocial, existential and information needs of patients and their lay and professional carers, and to review their use of health, social and voluntary services. Additionally, this study examined the utility of a qualitative longitudinal, multi‐perspective methodology in end‐of‐life research.	This was a feasibility trial of a complex intervention delivered by a supportive care liver nurse specialist which aimed to improve care coordination, anticipatory care planning and quality of life for people with advanced liver disease and their carers.	To assess the impact of early hospice intervention on patients' wellbeing and experience.
Participants (population)	Fourteen people with refractory ascites and eight nurses. There were 10 men with liver disease and four women with liver disease. Thirteen of the people with liver disease were White and one was Asian. Age range: 46–80 years. Of the nurses, all were female (age and ethnicity not provided).	In the relevant component study, there were nine men with cirrhosis and refractory ascites and three women. Two male carers and three female carers of people who had died from a complication of cirrhosis. Ages: people with liver disease were 40–79 years. Carers were 36–72 years old. Types of liver disease were alcohol related, nonalcoholic steatohepatitis and hepatocellular carcinoma.	Fifteen people with advanced liver disease: seven males and eight females. Age range: 35–84 years. Aetiologies: alcohol‐related, nonalcoholic fatty liver disease, hepatitis C, primary hepatocellular carcinoma, autoimmune hepatitis and cryptogenic liver disease. Eleven lay carers and 11 case‐linked professionals. The researcher acknowledges that people from ethnic minorities were missing from the study.	Forty‐seven people with advanced liver disease, who had an unplanned hospital admission with decompensated cirrhosis, were recruited. Thity‐one were men and 16 were women. Age range: 31–87 years. 31 had alcohol‐related, five had nonalcoholic fatty liver disease, 10 combination of causes and one unknown. *Thirty‐foue of these went on to receive the intervention*. Twenty‐seven family carers and 13 case‐linked professionals were recruited.	Twenty people with advanced liver disease. No other details are provided.
Methodological approach and study design, e.g., (1) phenomenology, (2) ethnography, (3) grounded theory, (4) case study.	This paper reported on the qualitative arm of the REDUCe study, which was a feasibility randomised control trial. Applied thematic analysis.	A qualitative approach was used in the component study with people who have liver disease and their carers (described in chapter 3). In the data analysis, a thematic approach was used. According to Hudson, this was directed towards both the organic development of theory, and development of existing knowledge. Elements of grounded theory were utilised (line‐by‐line coding, constant comparison, and memo‐writing) with pre‐existing and ‘a priori’ concepts included.	Qualitative. Constructivist grounded theory. Data were analysed with the patient and public involvement (PPI) group, ‘integrated case’ and longitudinally.	Feasibility trial with a mixed‐method evaluation. People with liver disease received a 6‐month intervention (alongside usual care) from a specially trained liver nurse specialist. Data analysis was thematic and informed by the PPI group.	Pilot project—model of joint working in the development of a shared care liver pathway established between a hospice and the hepatology team based at a district general hospital. Case studies.
Data collection method	Telephone interviews to explore the experiences, beliefs, and care pathways. The interview schedule was based on Dixon‐Woods' theoretical model of healthcare access.	Semistructured face‐to‐face interviews.	Serial face‐to‐face interviews (up to three times over 12 months).	Mixed methods. Case note analysis and questionnaires examined resource use, care planning processes and quality‐of‐life outcomes over time. Face‐to‐face or telephone interviews with patients, carers and professionals explored acceptability, effectiveness, feasibility and the intervention.	Monthly assessment of symptoms and wellbeing was adopted using the validated outcome measurement tools of palliative care outcome scale (POS) and POS‐symptoms (POS‐s).
Findings	A high level of acceptability for LTADs, particularly as they removed the need for hospital drainage. Benefits included personalised care, improved symptom control of ascites, and being at home. Furthermore, they facilitated regular engagement and support from community nursing teams that could help reduce social isolation and stigma. There was a consistent theme of people with liver disease wanting to avoid hospitalisation towards end of life.	The research found extensive physical, psychological and social burdens faced by patients with end‐stage liver disease and their families towards the end‐of‐life, and highlighted that their palliative care needs are frequently incompatible with the healthcare services available to address them.	This study identified uncertainty as the central pervasive factor in the experiences of patients, lay and professional carers. The needs of this patient group are currently poorly met from diagnosis to bereavement. Uncertainty makes advance care planning important, but difficult to know when to start.	The nurse‐led intervention was acceptable and feasible. Improvements to people's quality of life and care were demonstrated. People with liver disease, healthcare professionals and carers, welcomed access to additional expert advice, support and continuity of care.	Quinn et al.^32^ stated that this model of care addressed previously unmet holistic care needs, with timely interventions appearing to improve overall outcomes despite deteriorating health. Indications that a shared care pathway could reduce the length of stay and cost of paracentesis, as well as the number and length of hospital admissions.
Strengths and weaknesses	Strengths: This paper makes a valuable contribution—it is possibly the only qualitative study focusing on people's experience of LTAD in end‐stage liver disease. Weaknesses: details of PPI are lacking. Carers were not included. Lack of ethnic diversity among the participants.	Strengths: Hudson^33^ described this as the first study of patients and bereaved carers in end‐stage liver disease to focus on how existing services meet patient needs towards the end‐of‐life, ascertain perspectives on how such services could be improved pragmatically, and explore attitudes toward palliative care interventions directly. Weaknesses: PPI was not included. The ethnicity of the participants for the relevant component study is not mentioned.	Strengths: Kimbell^34^ described this as the first in‐depth serial interview study to explore people's experiences of advanced liver disease outside of transplantation. The longitudinal nature of the data collection allowed for the complex and evolving nature needs and experiences of patients, lay carers and professionals to be explored in more depth. Weaknesses: People from ethnic minorities were missing from the study.	Strengths: This was the first feasibility trial of integrating a palliative care approach within the role of a liver nurse specialist. The role of the PPI is clearly described. Weaknesses: described by Kimbell et al.^35^ ‘The study was limited to a small number of white patients, largely with alcohol‐related liver disease, attending one specialist centre in Scotland’. The intervention lasted only 6 months (p. 926).	Strengths: This paper has value because it illustrates an example of collaborative working and innovation to address issues relevant to people with advanced liver disease. Weaknesses: the paper has limitations because it was a pilot project and some details are missing, for example, ethical considerations and patients' characteristics. PPI is not mentioned.
Ethical considerations	Ethical approval was obtained from the National Research Ethics Committee in South Central Hampshire. Informed consent was obtained from participants. Confidentiality was maintained. The researchers also discussed ethical issues around the inclusion of people in the palliative stage of their illness having the opportunity to be involved in research.	Yes. Ethical approval was obtained from the Hampshire B NHS Research Ethics Committee. Ethical considerations are discussed in detail. Informed consent was obtained.	Ethics approval was obtained from South‐East Scotland Research Ethics Committee 1. Informed consent was obtained from participants. Ethics discussed in detail.	Ethical approval obtained from South‐East Scotland. Research Ethics Committee. They do not mention consent.	Not discussed.
PPI strategy	Not mentioned.	Not mentioned.	A patient and carer advisory group provided support and guidance throughout the project. The group supported data analysis.	A PPI with extensive involvement in advising on research in end‐of‐life collaborated in the design, conduct and evaluation of the study.	Not mentioned.
Relevant suggestions from the authors	The authors suggested that ‘qualitative data from the REDUCe study shows acceptance for an intervention to improve palliative management of ascites in ESLD’ (p. 323). They will be conducting a larger qualitative study as part of REDUCe‐2.	Hudson^33^ recommended studies to develop and prospectively evaluate specific palliative care interventions for people with end‐stage liver disease, and studies that focus on carers.	Kimbell^34^ argued that there is ‘an urgent need to ensure that people living and dying with advanced liver disease and their families benefit from appropriate, equitable and timely access to supportive and palliative care’ (p. 229).	Kimbell et al.^35^ recommended further studies assessing palliative care interventions for people with end‐stage liver disease, including randomised controlled trials, and qualitative research exploring carers' perspectives.	Quinn et al.^32^ stated that the next stage of the project will fully capture the implications and impact of hospice services for this group of patients. Both quantitative and qualitative data about the patients' and carers' lived experiences will help to inform future models of care. They also argued that further work is needed to explore the role of hospices with carers of patients with advanced liver disease.
Overall quality based on CASP^36^ checklist	High.	High	High	High	Moderate

Abbreviations: CASP, Critical Appraisal Skills Programme; REDUCe, refractory ascites due to cirrhosis.

### 2.5 Quality assessment

Assessment of the strengths and weaknesses of the literature was important to understanding how it could answer the research question. Papers were read, reread, and critically appraised.^37^ The Critical Appraisal Skills Programme^36^ checklist guided the quality assessment, with consideration of how the concepts of rigour, validity, and reliability in the research process were approached.^38^ Qualitative research is reflexive and subjective in nature, so these concepts are more suitable when assessing qualitative papers, as opposed to the idea of bias.^39^ The first reviewer (C. B.) undertook a quality assessment, with discussion and oversight from the rest of the review team. Four papers were assessed as high quality,^33^, ^34^, ^35^, ^40^ and one was medium quality.^32^

An additional criterion was included in the quality assessment process: PI in the research. This was essential to ensure that the studies were meaningful to people with liver disease and conducted *with* them rather than just *about* them.^41^ PI was not mentioned by Cooper et al,^40^ Hudson^33^ or Quinn et al.^32^ In contrast, Kimbell^34^ and Kimbell et al.^35^ had a clear PI strategy evidenced at different stages of the research process.

#### 2.5.1 Stage 4: Analyse

Reflexive thematic analysis was applied to data analysis^42^ and NVivo Pro 12.5 software was used to support this approach. Previous authors have found NVivo to be a rigorous way of organising the data to facilitate interpretation and to look for themes.^43^, ^44^ This involved the following process:

1. Papers were imported into NVivo Pro 12.5
2. Open Coding: Line‐by‐line coding of the results sections that were specifically relevant to answering the systematic review question.^43^ Results that did not answer the review question were not coded line by line. For example, in Kimbell's^34^ thesis, chapter seven was analysed in depth because it focused on individuals' experiences of care, whereas chapter five was not coded because it explored the onset, manifestation and understanding of the disease.
3. Words/phrases that reflected action were mainly used to create nodes in NVivo Pro 12.5 for example, *accessing hospital care*. This was to enable the author to keep an open mind, spark thinking, develop original ideas and avoid premature conceptualisation.^45^
4. Axial coding: Categories were developed from the open codes by looking at the connections and main concepts. For example, *information and communication*.
5. Selective coding: In this final stage, the overarching concepts were developed, specifically *disempowerment versus empowerment*, which are explored in the discussion section of this review.

#### 2.5.2 Stage 5: Present

## 3 RESULTS

Table 3 gives a summary of the demographics of individuals from the five studies. Analysis led to four main themes of experiences related to the following:

1. HCPs
2. Services
3. Support
4. Information and communication

**Table 3:** Summary of demographics.

Paper	Type of study	Females with liver disease	Males with liver disease	Age range of individuals	Ethnicity of individuals	Carers included
Cooper et al. (2021)^40^	Qualitative	4	10	46–80	13 White 1 Asian	0
Hudson (2019)^33^	Qualitative	3	9	40–79	Unreported	5
Kimbell (2014)^34^	Qualitative	8	7	35–84	White	11
Kimbell et al. (2018)^35^	Mixed methods	16^a^	31^a^	31–87	White	27
Quinn et al. (2017)^32^	Pilot study with 20 individuals	Unreported	Unreported	Unreported	Unreported	0
		Total females: 31	Total males: 57			Total carers: 43

^a^ Of the 47 people recruited for Kimbell et al.'s^35^ study, a total of 34 individuals went on to receive the intervention.

These themes incorporated aspects that were either disempowering or empowering to individuals with liver disease, and person‐centred care was found to be an important aspect of positive experiences.

Theme 1 Experiences as related to HCPs

There were frequent references to interactions and relationships with a range of HCPs throughout the data, with particular emphasis on the role of general practitioners (GPs) and specialist nurses. Conflicting experiences and perspectives of HCPs were apparent, depending on service structures and HCPs' communication skills. Clinicians who were responsive, well‐informed and person‐centred improved care experiences.

### 3.1 Perceptions of GPs in liver disease care

GPs were commonly cited, except in Quinn et al.^32^ People with liver disease expressed mixed experiences of their GP's involvement, ranging from supportive and actively interested,^34^ to lacking confidence and knowledge about liver disease, tending to leave decision‐making to specialists.^33^, ^34^ GPs were sometimes the first port‐of‐call for a person with liver disease, for example, to access paracentesis,^40^ for psychological issues, or when dying at home.^34^ There was variation in how supportive GPs were perceived to be, with their contribution improved when they were more informed, supported, and care was co‐ordinated, as illustrated by Kimbell et al.'s^35^ study in which the specialist nurse engaged with GPs to arrange advanced care plans.

### 3.2 Specialist nurses

The importance of specialist nurses was cited as facilitating a person‐centred approach through care coordination, information, and support to people with liver disease, their carers, and other HCPs. This was particularly evident in Kimbell et al.'s^35^ feasibility trial of a supportive care liver nurse specialist for people with liver disease. Empowering experiences were described: ‘We felt we were getting more answers, whereas in the hospital you feel as if it is, ‘We're too busy, we've got other patients, you're not the only patient, we've got this patient to see’. […] When [study nurse] was here we got a chance to speak. It was good, we did get a lot of feedback; it was really helpful’.^35^ ^, p.923^ Similar accounts were evident regarding an alcohol liaison nurse^34^ and collaboration between the hospital and hospice specialist nurses.^32^ In Hudson's^33^ research, the essential role of hepatology specialist nurses for coordination of care and advice was described but with frustration if the nurse was unavailable. It was not only *specialist* nurses who were cited positively, in Cooper et al.,^40^ regular home visits from community nurses provided valuable emotional support. In Kimbell,^34^ there were encouraging descriptions of hospital staff, but there were contrasting examples of people with liver disease feeling mistreated by HCPs.

Theme 2 Experiences of services

There was a theme of experiences of healthcare services being empowering or disempowering to individuals with liver disease depending on:

1. Where care took place—primarily within secondary care, with exceptions depending on the structure of local services.
2. Access to services.
3. Access to specialist and palliative and end‐of‐life care.

### 3.3 Hospital

It was apparent that hospitals played a huge role in how liver disease was managed towards the end of life, with frequent admissions, prolonged stays, and regular appointments being described as part of people's experiences. Sometimes this was positive and empowering for people who were so familiar with the wards and staff that it was like coming home when they were admitted to hospital.^34^ There were many examples of satisfaction with the care and support people received, including the suggestion from carers of those who had died in the hospital that they did not regret this experience.^34^ However, overall people wanted to avoid it whenever possible: ‘I did not like going into hospital’,^40^ ^, p.319^ and ‘I hate being in hospital. Everyone uses all these long words and it is scary and I just want to get out’.^33^ ^, p.129^

Disempowering factors included a distressing environment with a lack of privacy, other patients, lengthy stays, waiting around, and examples of poor communication from HCPs. In contrast, despite initial apprehension about hospice care, individuals in Quinn et al.'s^32^ project appreciated the calming environment of the hospice compared with a busy outpatient setting. People in Cooper et al.'s^40^ study had reduced hospital admissions and had much of their care provided at home when they had a long‐term abdominal drain in situ.

### 3.4 Accessing services

Mixed experiences of accessing healthcare services for people with liver disease were apparent, impacting satisfaction, well‐being, and sense of empowerment as illustrated in Table 4. Except in Kimbell et al.,^35^ an important part of care experiences for people with ascites was access to paracentesis, relating to the type of drain and where drainage occurred. Although paracentesis is a form of symptom relief, rather than active treatment, it was not always perceived this way by individuals with ascites who sometimes viewed it as prolonging life.^34^, ^40^

**Table 4:** Experiences of accessing services.

Negative experiences of accessing healthcare services	Positive experiences of accessing healthcare services
Organisational challenges or indirect access for people trying to access paracentesis in a hospital.^40^ The location of services, for example, being far from home. Having to travel to services. Parking challenges. Financial issues (related to parking, travel costs, or relatives giving up work time to support).^33^ The impact on friends/family supporting people with liver disease to get to appointments.^33^, ^34^	Telephone access, for example, to advice/support,^35^ or direct access to departments for paracentesis.^40^ Services in a convenient location, for example, person's home^35^, ^40^ or their nearest hospital.^34^ Preferred environment, for example, hospice.^32^ Co‐ordinated care, for example, the role of specialist nurses^35^ and the hospice^32^ facilitating access to services.

### 3.5 Access to specialist palliative and end‐of‐life care

A wider issue was the discrepancy in access to palliative care and services specifically for people towards the end of life. People with liver disease rarely accessed hospices, except in Quinn et al.^32^ Despite long‐term abdominal drains in Cooper et al.'s^40^ study being described as palliative, it was unclear what other palliative services individuals had access to, and it was suggested that some did not recognise their care as palliative. There was little mention of allied health professionals in the papers, which is significant because the high physical and psychological symptom burden of advanced liver disease was clear. The limited reference to other professionals was indicative that individuals often did not receive appropriate palliative support. Exceptions to this included the hospice's coordinated approach to providing supportive services^32^ and the supportive care liver nurse specialist.^35^

Theme 3 Experiences of support for people with liver disease

People with liver disease, and their carers, received support from a variety of sources, with barriers and facilitators apparent, as illustrated in Table 5.

**Table 5:** Experiences of barriers and facilitators to support.

Experiences of barriers to support	Experiences of facilitators to support
The structure of services, for example, in Cooper et al.'s^40^ study, people sometimes had challenges accessing paracentesis. Not knowing how to access support, for example, how to contact consultants for advice.^34^ Poor care/communication from HCPs.^34^ Service constraints, for example, time‐limited appointments.^33^, ^34^	Regular contact, for example, visits from community nurses.^40^ Easy access, for example, to telephone support.^35^ Having a positive relationship with a HCP, for example,, ‘Several participants had a good relationship with their GP, who would visit them at home to check on their health, and considered them approachable and supportive’.^34^ ^, p.144^ Access to specialist nurses such as the supportive care liver nurse specialist,^35^ and the alcohol liaison nurse.^34^ Knowing where to go for support.^35^ Co‐ordinated care, for example, the role of the hospice.^32^ Having supportive friends and family.^34^

Abbreviations: GP, general practitioner; HCP, healthcare proffesional.

### 3.6 The role of carers

There was a reference to family and friends having a vital role as carers, including taking people to appointments, supporting them with care coordination and access, and liaising with HCPs. Sometimes this was challenging, causing stress and deteriorations in carers' own physical health and emotional wellbeing.^33^

Theme 4 Information and communication

The concepts of information and communication were interrelated in the papers. Information, or lack of it, emerged from the data, and *how* this information was communicated, or not communicated was relevant. For example, people with liver disease did not always know what questions to ask but having a specialist nurse who gave information and was available to answer their questions in an accessible way was empowering in Kimbell et al.'s^35^ study. Sometimes, people were disempowered and dissatisfied with the information they received and/or the way it was communicated to them.^33^, ^34^ Occasionally, there existed a mismatch between the information that HCPs provided and the information that people with liver disease wanted to receive: ‘I told them that, I want you to tell me the truth, no holds barred like, but they couldn't answer it. Because they didn't know’^33^ ^, p.122^ and ‘I remember asking the London doctor if there was anything he could do about it (my fatigue and itch)—he just said yes there is but he just needed to get the tests done first. He's never said more than that’.^33^ ^, p.131^. Lack of time for effective communication was described: ‘You just get the feeling that the doctors are unapproachable because they're so busy’.^34^ ^, p.148^

### 3.7 Prognosis

People with liver disease regularly lacked information and knowledge about their prognosis and what to expect for a range of reasons:

1. Uncertain disease trajectory—the unpredictable nature of liver disease.
2. HCPs not knowing/understanding the prognosis or having the confidence to have conversations with individuals about these issues.
3. People with liver disease not knowing what questions to ask.
4. People with liver disease not wanting to lose hope.^33^, ^34^

*How* information was communicated was important. For example, an individual who stopped seeing her GP because ‘She was just so negative, and I found it really … I want them to be honest but I don't want them to, you know, be so negative’.^34^ ^, p.144^ Empowering examples included: ‘Although the hospice can't cure my illness, it has helped me to cope with it. I feel less stressed and more confident having learned how to manage my energy and being aware of my limitations. My experience at the hospice has turned my life around’.^32^ ^, p.S20^ This was not typical within the results and Hudson^33^ found that individuals with liver disease were often unsure of the implications of their diagnosis, with persistent uncertainty disempowering them from making plans.

## 4 DISCUSSION

In this review, only five studies (detailed in Table 2) met the inclusion criteria to explore the experiences of people with liver disease in palliative and end‐of‐life care in the United Kingdom, confirming that this is an under‐researched area.^16^ There was a lack of ethnic diversity in the individuals included in the studies, with the majority being white. More males participated in the studies, but this reflects the higher number of men who have liver disease compared with women.^2^ Experiences were found to depend on who provided care and the structure of local services. There were variations in what care and support were available and where these were provided in terms of the person's home, clinics, hospital, and hospice, with most care taking place in secondary care. There were examples of person‐centred approaches, but overall, there was a lack of advanced care planning, accessible information, and early referral to palliative care services. Crucially, people with advanced liver disease were often unclear about their illness and prognosis, meaning that their experiences were not necessarily that of care, which they perceived as palliative or end‐of‐life. This is not unique to liver disease: Gurgenci and Good^46^ found that internationally, people with end‐stage cardiac failure lacked information and advanced care planning, facing uncertainty and delayed access to palliative care.

### 4.1 Disempowerment versus empowerment

In healthcare, empowerment has been described as ‘a process through which people gain greater control over decisions and actions affecting their health’.^47^ In contrast, disempowerment is having a lack of control over one's life and no power to change things.^48^ Distinct features of empowerment have been found for people with advanced life‐limiting illnesses, relating to physical and psychosocial challenges, and Wakefield et al.^49^ argued for greater efforts to progress the empowerment of people approaching the end of their lives.^49^

An overarching feature of this review was the presence of disempowering versus empowering experiences for people with liver disease in palliative and end‐of‐life care. Disempowerment was apparent when

1. Relationships with HCPs were dissatisfactory.
2. Services were mismatched with the needs of people with liver disease.
3. There were challenges in accessing care/support.
4. Information/communication for people with liver disease was inadequate.

Other researchers have illustrated disempowerment facing people with advanced liver disease, arguing that this is caused by a complex interaction of limited resources, lack of HCP training and confidence in raising issues regarding the end of life.^30^ HCPs may themselves be disempowered to appropriately support people with liver disease, due to lack of confidence, skills and knowledge about palliative care.^4^ Greater understanding of the emotional needs of people with advanced liver disease has been advocated as a means of empowering HCPs to provide good quality palliative care.^50^

In this review, empowering experiences were apparent when there were

1. Positive relationships with HCPs.
2. Services designed to meet palliative and end‐of‐life care needs.
3. Accessible treatment, support, and advice.
4. Appropriate information communicated in a sensitive way to people with liver disease and their carers.

### 4.2 Person‐centred care and empowerment

Internationally, researchers have advocated for liver disease services to be more person‐centred.^51^ Where services are specifically designed to be person‐centred, there is an opportunity for individuals, carers, and HCPs to have more empowering experiences—being empowered to share knowledge, work collaboratively, and make decisions. In this review, there were examples of services designed to improve the palliative and end‐of‐life care experiences of people with advanced liver disease, and the people who support them.^32^, ^35^, ^40^ Similarly, in Chivinge et al.'s^27^ service improvement project, a nurse‐led paracentesis service improved patient satisfaction and reduced complaints and waiting times. The perspectives of carers supporting people with advanced liver disease are also important to understanding care experiences and facilitating person‐centred palliative care.^52^ Experiences are improved when there is a collaboration between hepatology, community services, and specialist palliative care, and there is advanced care planning.^32^, ^35^, ^52^ Researchers of other specialisms have also advocated person‐centred care as a means of empowering people with long‐term health conditions. For example, a Finnish study with people who have type 2 diabetes found that person‐centred care was associated with higher empowerment levels.^53^

Tackling healthcare inequalities has been described as a prime focus for action^18^ and person‐centred care is a vital part of this. Without consideration of the needs of individuals and communities, inequalities persist. Appropriate palliative and end‐of‐life care services for everyone with advanced liver disease are important to addressing healthcare inequalities and empowering people towards the end of their lives.

### 4.3 Public involvement

It is significant that PI was missing from Quinn et al.,^32^ Hudson,^33^ and Cooper et al.,^40^ because as illustrated by Kimbell^34^ and Kimbell et al.,^35^ PI can facilitate empowerment by providing the opportunity to influence research that is relevant to people with liver disease.^54^ Studies that embed PI in the research design are recommended to ensure they are meaningful, inclusive, and collaborative with people affected by liver disease.^55^ As part of the systematic review process, an online PI workshop organised through Voice^56^ was held with four people who have liver disease and four people who are carers for people with liver disease. The lead reviewer (C. B.) presented the findings and discussed them with the group who offered their perspectives:

There is a stigma attached to liver disease which causes problems in communication between patients, medical professionals, etc.

Would fully endorse your findings. The information given is poor… The toll on carers is enormous but the quality of care is highly dependent on how well they are educated.

It is important for people near the end of life to not be isolated. They need to be brought into the community more but because of lack of funding currently, this is becoming more difficult.

The need for research to explore the perspectives of people from ethnically diverse backgrounds was also highlighted by the PI group, especially to understand the cultural needs of individuals with liver disease and their families towards the end of life.

### 4.4 Strengths and limitations of the review

To the authors' knowledge, this is the first systematic review to focus on the care experiences of people with liver disease in palliative and end‐of‐life care in the United Kingdom. Wolfswinkel et al.'s^21^ five‐step approach facilitated a rigorous method for the review process and theory development. Using NVivo 12.5 software was an efficient way of organising the data, supporting thorough analysis and theme development. The PI workshop was valuable, adding credibility to the findings and could have been beneficial at earlier stages of the review process.^57^

In conducting this review, it was challenging to differentiate between care experienced by people with liver disease as palliative and/or end‐of‐life, and care experienced as active treatment. This was for the following reasons:

1. The unpredictable trajectory of liver disease.
2. The language used to describe care by authors and people participating in the studies.
3. Uncertainty (in individuals and HCPs).

In the review papers, it was sometimes unclear when liver disease care was end‐of‐life. For example, in Kimbell's^34^ research, nine of the 15 participants with advanced liver disease died by the end of the yearlong study. However, six did not die and two individuals had an improvement in their health. People with liver disease may themselves be unclear when the care they are experiencing is palliative and/or end‐of‐life, as illustrated by some individuals' perception of palliative long‐term abdominal drains as prolonging life,^40^ and prognoses being rarely discussed.^34^ Other researchers have commented that people with advanced liver disease may not discuss their illness as being palliative, even when their prognosis is poor.^29^ HCPs can be uncertain about this too.^4^ For these reasons, when exploring end of life and palliative care experiences, researchers must pay careful attention to the language used when conducting research with people who have advanced liver disease.

## 5 CONCLUSION

This review has found that people with advanced liver disease have mixed experiences of palliative and end‐of‐life care, depending on the quality of relationships with HCPs, the structure of local services, support available to them, the provision of information and how this is communicated to them. These factors contribute to whether care experiences are empowering or disempowering to individuals, with the concept of person‐centred care identified as an important consideration. There is a need for further research with a more diverse population beyond a sample of predominantly white people. PI should be embedded into the design, to further explore the perspectives of individuals with advanced liver disease and their carers regarding palliative and end‐of‐life care specifically, but with recognition that people with liver disease and their supporters may sometimes be unclear when care *is* palliative or end‐of‐life.

## CONFLICT OF INTEREST STATEMENT

The authors declare no conflict of interest.

## Supporting information

Supporting information.

## Data Availability

The Data that support the findings of this study are available from the corresponding author upon reasonable request.
