# Supplementary material for: The experiences of people with liver disease of palliative and end‐of‐life care in the United Kingdom—A systematic literature review and metasynthesis
Source: Health Expect. 2023 Oct 19;27(1):e13893. doi: 10.1111/hex.13893 (PMC10768859; doi:10.1111/hex.13893)
Supplement: Supplementary file 1 — Supporting information. [file HEX-27-e13893-s001.docx]

# Appendix 1.

Adapted from the ACTIVE Framework (Pollock et al. 2019) to describe stakeholder involvement in systematic reviews

| **Framework constructs** | **Categories** |
| --- | --- |
| Who was involved? | People with liver disease and carers:   1. 39-year-old woman, British Asian, has liver disease. 2. 42-year-old woman, British Asian, has liver disease. 3. 73-year-old man, White British, has liver disease. 4. 68-year-old woman, White British, has liver disease. 5. 42-year-old woman, Asian British, carer for relative with liver disease. 6. 69-year-old woman, White British, carer for relative with liver disease. 7. 73-year-old woman, White British, carer for relative with liver disease. 8. Age and ethnicity not provided. Woman who is a carer for her relative with liver disease. |
| How were they recruited? | Advertised on Voice (Voice 2023) |
| What was the mode of involvement? | A 90-minute online workshop facilitated by the lead reviewer to discuss the findings of the review, and to contribute to the development of a research study exploring people’s experiences of care in advanced liver disease. A further meeting is planned for later in 2023. |
| At what stage of the review process did involvement occur? | After data analysis, whilst this paper was being written up. The group were provided with information about the findings and contributed their knowledge, experience and perspectives. |

Pollock, A., Campbell, P., Struthers, C., Synnot, A., Nunn, J., Hill, S., Goodare, H., Morris, J., Watts, C. and Morley, R., 2019. Development of the ACTIVE framework to describe stakeholder involvement in systematic reviews. *Journal of Health Services Research & Policy*, 24 (4), 245-255.

Voice, 2023. *Browse: The opportunities, groups and news, from across the UK, updated daily.* [online]. Avilable from: <https://voice-global.org/> [Accessed 1st April 2023].
